# Cost-effectiveness of intensive multifactorial treatment compared with routine care for individuals with screen-detected Type 2 diabetes: analysis of the ADDITION-UK cluster-randomized controlled trial

**DOI:** 10.1111/dme.12711

**Published:** 2015-04-15

**Authors:** L Tao, E C F Wilson, N J Wareham, A Sandbæk, G E H M Rutten, T Lauritzen, K Khunti, M J Davies, K Borch-Johnsen, S J Griffin, R K Simmons

**Affiliations:** 1 MRC Epidemiology Unit, University of CambridgeNorwich, UK; 2 Cambridge Centre for Health Services Research, University of CambridgeNorwich, UK; 3 Health Economics Group, University of East AngliaNorwich, UK; 4 Department of Public Health, Section of General Practice, University of AarhusDenmark; 5 Julius Center for Health Sciences and Primary Care, University Medical Center UtrechtThe Netherlands; 6 Diabetes Research Centre, University of LeicesterUK; 7 Holbæk HospitalDenmark

## Abstract

**Aims:**

To examine the short- and long-term cost-effectiveness of intensive multifactorial treatment compared with routine care among people with screen-detected Type 2 diabetes.

**Methods:**

Cost–utility analysis in ADDITION-UK, a cluster-randomized controlled trial of early intensive treatment in people with screen-detected diabetes in 69 UK general practices. Unit treatment costs and utility decrement data were taken from published literature. Accumulated costs and quality-adjusted life years (QALYs) were calculated using ADDITION-UK data from 1 to 5 years (short-term analysis, *n* = 1024); trial data were extrapolated to 30 years using the UKPDS outcomes model (version 1.3) (long-term analysis; *n* = 999). All costs were transformed to the UK 2009/10 price level.

**Results:**

Adjusted incremental costs to the NHS were £285, £935, £1190 and £1745 over a 1-, 5-, 10- and 30-year time horizon, respectively (discounted at 3.5%). Adjusted incremental QALYs were 0.0000, – 0.0040, 0.0140 and 0.0465 over the same time horizons. Point estimate incremental cost-effectiveness ratios (ICERs) suggested that the intervention was not cost-effective although the ratio improved over time: the ICER over 10 years was £82 250, falling to £37 500 over 30 years. The ICER fell below £30 000 only when the intervention cost was below £631 per patient: we estimated the cost at £981.

**Conclusion:**

Given conventional thresholds of cost-effectiveness, the intensive treatment delivered in ADDITION was not cost-effective compared with routine care for individuals with screen-detected diabetes in the UK. The intervention may be cost-effective if it can be delivered at reduced cost.

## Introduction

Type 2 diabetes is associated with increased risk of costly macro- and microvascular complications [1,2]. Treating diabetes accounts for over 10% of the UK National Health Service (NHS) budget, and the condition exerts a heavy burden on people with diabetes and on those who care for them [3].

There are a number of treatment options for individuals with established Type 2 diabetes. National Institute for Health and Care Excellence (NICE) guidelines recommend structured lifestyle interventions focusing on diet, physical activity and smoking cessation as the first line of treatment, followed by pharmacological management of cardiovascular risk factors [4–6]. Intensive multifactorial treatment of individuals with established diabetes reduces the risk of cardiovascular events and premature death by 50% and is cost-effective relative to other preventive interventions [7,8]. With the advent of national programmes, such as the UK Health Checks [9,10], many people will be diagnosed with diabetes earlier in the disease trajectory. Less is known about the effectiveness and cost-effectiveness of intensive treatment among these newly diagnosed patients. The balance of benefits, harms and costs of intensive treatment may be different among individuals with screen-detected diabetes compared with those with clinically diagnosed and long-standing diabetes.

**What's new?**

- Existing evidence suggests that intensive multifactorial treatment of individuals with established diabetes reduces the risk of cardiovascular events by 50% and is cost-effective relative to other preventive interventions.
- Less is known about the cost-effectiveness of treatment earlier in the disease trajectory.
- Under conventional thresholds of cost-effectiveness, interventions to promote intensive multifactorial treatment were not cost-effective compared with routine care for individuals with screen-detected diabetes in the UK.

The ADDITION–Europe study was a cluster-randomized trial of the effect of intensive multifactorial treatment compared with routine care on cardiovascular risk over 5 years among individuals with screen-detected Type 2 diabetes [11]. The intervention was associated with small, but significant increases in cardioprotective treatment, and in turn, a non-significant 17% relative risk reduction in the incidence of a composite cardiovascular endpoint [12]. We report the short-term (1- to 6-year within-trial analysis) and long-term (10–30 years based on decision modelling) cost-effectiveness of the intervention in the UK from a UK payer (NHS) perspective.

## Methods

The ADDITION–Europe trial (NCT 00237549) consisted of two phases – a screening programme and a pragmatic cluster-randomized controlled trial comparing the effects of intensive multifactorial therapy with routine care among individuals with screen-detected Type 2 diabetes [11,12]. This analysis used data from the two UK centres (Cambridge and Leicester) included in the ADDITION trial. Briefly, participants aged 40–69 years, without known diabetes registered with 69 general practices were invited to stepwise screening. In total, 1026 (Cambridge 867 and Leicester 159) eligible participants with screen-detected diabetes agreed to take part in the treatment phase of the trial. General practitioners (GPs) excluded those with an illness with a life expectancy of less than 12 months, or who were housebound, pregnant or lactating, or with psychological or psychiatric problems that were likely to invalidate informed consent. This analysis focused on the treatment phase of the trial. Participants were treated according to the group to which their practice had been allocated: intensive treatment or routine care. Group allocation was concealed from participants throughout the trial. The study was approved by local ethics committees in each centre. All participants provided written informed consent.

### Description of comparators

In the routine care group, participants with screen-detected diabetes received usual diabetes care through the UK NHS according to current recommendations [13–15]. Practices received additional funding equivalent to two 10-min and one 30-min consultation with a GP and four 15-min nurse appointments per patient per year for 3 years. These are routine NHS care costs and are not therefore included in the cost-effectiveness analysis.

In the intensive treatment group, additional features were added to educate and support GPs, practice nurses and participants in target-driven management of diabetes. The intervention was delivered in general practice in Cambridge and in peripatetic community clinics in Leicester. Detailed information can be viewed on the ADDITION website (http://www.addition.au.dk).

Briefly, in Cambridge, the intensification of diabetes management was promoted through the addition of a number of features to existing diabetes care. These included funding to facilitate more frequent contact between patients and practitioners and a practice-based academic detailing session conducted by a local diabetologist and a GP opinion leader. Interactive practice-based audit and feedback sessions were organized around 6 and 14 months after the initial education session and annually thereafter. Practice nurses were provided with theory-based education materials to give to participants in order to provide a shared framework for discussion of the causes, consequences and treatment of diabetes. Participants were encouraged to lose weight, increase their physical activity, avoid excessive alcohol intake, take their medication regularly, self-monitor their blood glucose level if given a glucometer by their practice, and attend annual health checks. Participants who smoked were encouraged to stop. GPs were also recommended to refer patients to a dietician.

In Leicester, care was organized and delivered by a core team of specialist practitioners (consisting of a doctor, nurse and dietician) within peripatetic community clinics. Early participation in structured education for newly diagnosed people with Type 2 diabetes (the DESMOND programme [16]) was encouraged and dietary support offered. DESMOND is a group education programme delivered by registered healthcare professionals, supported by a quality assurance component of internal and external assessment to ensure consistency of delivery. The programme is 6 h long, deliverable in either one full day or two half-day equivalents, and facilitated by two educators. Learning is elicited rather than taught, and most of the curriculum focuses on lifestyle factors, such as food choices, physical activity and cardiovascular risk factors. The programme encourages participants to consider their own personal risk factors and, in keeping with theories of self-efficacy, to choose a specific achievable goal to work on.

For both centres, treatment algorithms were based on trial data demonstrating the benefits of intensive treatment of cardiovascular risk factors in people with diabetes [8,17,18]. GPs were advised to consider prescribing an angiotensin-converting enzyme inhibitor to participants with a blood pressure ≥ 120/80 mmHg and a previous cardiovascular event or at least one other cardiovascular risk factor [17]. The remainder of the intervention was based on the stepwise regimen from the Steno–2 study [8] aimed at optimizing hyperglycaemia, hypertension, dyslipidaemia and microalbuminuria. GPs were also advised to consider prescribing 75 mg of aspirin daily to all patients without specific contraindications. The intensive treatment protocol was revised after publication of the Heart Protection Study [19] to include a recommendation to prescribe a statin to all individuals with a cholesterol level of ≥ 3.5 mmol/l.

### Measurement and endpoints

Health assessments at baseline and 5 years included biochemical, anthropometric and questionnaire measures, and were undertaken by centrally trained staff following standard operating procedures blind to study group allocation. Standardized self-report questionnaires were used to collect information on sociodemographic characteristics (education, employment, and ethnicity) and lifestyle habits (smoking status, alcohol consumption). Changes in biochemical measures and medication from baseline to 5-year follow-up have been reported previously [12]. Individuals were followed for a mean of 5.0 (sd 1.1) years. The primary outcome was time to cardiovascular event after diagnosis of diabetes, including cardiovascular mortality, cardiovascular morbidity (non-fatal myocardial infarction and non-fatal stroke), revascularization and non-traumatic amputation. All events were independently adjudicated by two members of a local endpoint steering committee, blind to group allocation according to an agreed protocol using standardized case report forms.

### Costs

Costing comprised the cost of delivering the intervention itself plus the routine cost to the NHS of treating diabetes and diabetes-related events observed in the trial. All costs were calculated in GBP (£) and monetary values were transformed to the 2009/10 UK national level using the Hospital & Community Health Services (HCHS) Pay and Prices Index [20,21].

### Cost of delivering the ADDITION intervention

Costs of delivering the intervention included: (1) materials, encompassing design, consultation meetings with health professionals regarding development and production; (2) practitioner and patient meetings, which included the costs of delivering the meetings, consultant and educator time, and doctors and nurse time; and (3) extra patient consultations and treatment (including prescription of cardio-protective medication and glucometers with strips, Table 1). The unit cost of doctor, nurse and other health professional time was obtained from standard UK unit cost references [20,21]. The volume of resources used was obtained from the ADDITION study protocol and relevant trial documents. Some costs were estimated from internal accounting during the trial. The cost for extra prescriptions in the intensive treatment group (compared with the routine care group) was established in treatment algorithms in 2001 at the beginning of ADDITION study as compensation to GPs and was transformed to 2009/10 prices. Intervention costs were different in Cambridge and Leicester and were averaged for the purposes of the cost-effectiveness analysis. For the long-term analysis, we assumed the additional prescription costs in the intervention arm would continue to be incurred each year.

**Table 1:** Cost of delivering intensive treatment in the UK in the five years following diagnosis (£, 2009/10 UK national level, discounted at 3.5%)

Category			Personnel/item	Time/times	Others	Unit cost (£)	Cost (£)	Remark
Delivery	Materials	Design	2 associated health professionals	8 h	–	44*	704	
		Practitioner folders	118 doctors and 52 nurses	–	–	8.2	1 394	Internal accounting
		Patient folders	513 patients (intensive treatment group)	–	–	5.8	2 975	Internal accounting
		Hand-outs	–	–	–	–	446	Internal accounting
		Consultation meeting	1 associated health professional (and 6 patients)	2 h	£5 for travel	44*	93	Patient cost excluded
		Focus group meeting	3 consultants	3 h	£5 for travel	121†	1104	
			3 nurses	3 h	£5 for travel	44*	411	
	Preparatory meetings	Cambridge (in GPs)	2 consultants	3 h × 100		121†	72 600	
			4.5 doctors	1.5 h × 100		121†	81 675	
			2 nurses	1.5 h × 100		30‡	9 000	
		Leicester (in hospitals)	2 educators	6 h × 6	£54.1 for logistics × 6	44*	3 493	
**Sub-total**							**173 895**	
Extra consultations		Cambridge (452)	10 min GP visit	3 times × 3 years		185§	121 091	Annual discount rate=3.5%
			10 min nurse visit	3 times × 3 years		30‡	19 637	
		Leicester (61)	GP initial visit + GP extra visits + annual review visit	60 min + 20 min × 4 + 30 min		165¶^,^**	28 534	
			2 visits + annual review visit	(20 min + 30 min) × 4		165¶^,^**	20 748	
**Sub-total**							**190 010**	
Extra treatments		Cambridge (452)	Extra prescription of sulfonylureas (SU), angiotensin-converting enzyme inhibitors and statins		Cost = £262.5 per patient	262.5	118 650	Internal accounting
		Leicester (61)	Extra prescription of SU, angiotensin-converting enzyme inhibitors and statins		Cost = £262.5 per patient	262.5	16 013	Internal accounting
			95% of participants issued with a glucometer and box of 50 strips at diagnosis		Glucometer cost = £1438 Strips cost = £2968	4 406	4 406	Internal accounting
**Sub-total**							**139 069**	
Total							502 974	
Cost/person							981	

All costs came from PSSRU unit costs of health and social care 2010 [21].

* Cost of advanced nurse time (includes lead specialist, clinical nurse specialist and senior specialist) per hour: £44 (Source: per hour of advanced nurse time, with qualification costs, table 10.7).

† Cost of health professional or consultant per hour: £121 (Source: per hour of GMS activity, with qualification costs, table 10.8b).

‡ Cost per GP nurse or research assistant hour: £30 [Source: per hour of nurse (GP practice), with qualification costs, table 10.6].

§ Cost of GP patient contact hour: £185 (Source: per hour of GP patient contact, with qualification costs, table 10.8b).

¶ Cost per hour of hospital nurse: £52 [Source: per hour of nurse 24-h (includes staff nurse, registered nurse, registered practitioner) patient contact, with qualification costs, table 14.4].

** Cost of hospital doctor patient contact hour: £169 (Source: per hour of consultant medical patient contact, with qualification costs, table 15.5).

### Cost of treatment of diabetes and diabetes-related complication treatment

Unit treatment costs were obtained from published literature (Table 2). We collected the annual treatment cost of Type 2 diabetes without complications and Type 2 diabetes-related complications, in the year of the event and in subsequent years. We counted both inpatient (cost of admissions to hospital either as a day case or as an inpatient for one or more nights) and non-inpatient costs (cost of all home, clinic and telephone contacts with GPs, nurses, podiatrists, opticians and dieticians, and with eye and other hospital outpatient clinics) from the UK Prospective Diabetes Study (UKPDS) [22] from which the majority of treatment costs used in this study were taken. In the short-term within-in trial cost-effectiveness analysis and the long-term modelling analysis we used an additive method to sum the annual costs of multiple complications.

**Table 2:** Unit cost (£, 2009/10 UK national level) and utility decrement for diabetes and diabetic complications

	Year of event		Subsequent years			
	Fatal	Non-fatal		Ref.	Utility decrement	Ref.
Type 2 diabetes	–	494.5	494.5	[22]	–0.220	[45]
IHD	–	3 558.4	1 175.2	[22]	–0.090	[24]
MI	2 295.6	6 861.8	1 129.8	[22]	–0.055	[24]
Heart failure	3 968.4	3 968.4	1 391.1	[22]	–0.108	[24]
Stroke	5 786.8	4 196.9	793.4	[22]	–0.164	[24]
Revascularisation	–	4 943.1	316.3	[46]	–0.059	[46]
Amputation	13 664.2	13 664.2	788.7	[22]	–0.280	[24]
Blindness	–	1 791.7	758.9	[22]	–0.074	[24]
Renal failure	30 599.2	30 599.2	30 599.2	[45]	–0.263	[24]
CVD death	3 724.3	–	–	[46]	–	

Costs extracted from the UKPDS study were based on participant hospital records and survey of 3488 UKPDS participants in 1996–97 from which inpatient and outpatient costs were predicted [22] and updated to 2009/10 price year.

### Utility decrement

We collected published utility decrement data to calculate quality-adjusted life years (QALYs) as the health outcome measurement for diabetes without complications and diabetes with complications including ischaemic heart disease, myocardial infarction, heart failure, stroke, revascularization, amputation, blindness and renal failure, from published literature **(**Table 2**)** [24]. The majority of these utility data were taken from the UKPDS based on EQ5D measurement [25]. The same value was assigned to the year of the event and for subsequent years. For patients with multiple events, the additive method was used where we summed utility decrements from each event.

### Short-term cost-effectiveness analysis

We calculated the accumulated costs and QALYs for every year from diabetes diagnosis based on observed events (myocardial infarction, stroke, revascularization and amputation) in the ADDITION trial. Both costs and QALYs incurred after the first year were discounted at 3.5% per annum, in line with current UK guidelines [26]. In order to adjust for baseline imbalances, we used ordinary least squares regression analyses to calculate cost and QALYs as a function of intervention group (routine care/intensive treatment), treatment centre (Cambridge/Leicester), age at diagnosis, gender and HbA_1c_ at baseline. Adjusted incremental treatment costs and QALYs were reported as means and 95% confidence intervals.

### Long-term modelling cost-effectiveness analysis

We used the UKPDS outcomes model (version 1.3) to perform long-term modelling analysis [27], because it is derived from a UK population and focuses on cardiovascular complications. We previously undertook a validation analysis [28] and concluded that the model provided a reasonable prediction of the incremental event rate although the UKPDS model tended to overestimate the absolute event rates.

The UKPDS outcomes model predicts future events year by year, based on a series of risk equations derived from the initial UKPDS cohort: in general, risk equations to predict whether an event occurs in year *t* are a function of the baseline value, years since diagnosis of diabetes and the value of the risk factor in the previous year [23]. Information from *ADDITION* trial participants at baseline was entered into the UKPDS outcomes model including age at diagnosis, sex, ethnicity, duration of diabetes, weight, height, smoking status, systolic blood pressure, HbA_1c_, total cholesterol, HDL-cholesterol and years since pre-existing CVD events. Values of smoking status, systolic blood pressure, HbA_1c_, total-cholesterol and HDL-cholesterol were also included from measurements taken at one and five-year follow-up. For the years in between (2, 3 and 4) and for future years, the risk factor values were simulated by the UKPDS outcomes risk equations, i.e. left to propagate through the long-term model. Data on atrial fibrillation, peripheral vascular disease, ischemic heart disease, congestive heart failure, amputation, blindness and renal failure at diagnosis were not collected in the ADDITION study. Given that all participants were newly diagnosed, all values were set to zero for these variables.

To deal with missing data in ADDITION-UK, multiple imputation was applied using the Markov chain Monte Carlo method assuming an arbitrary missing pattern [29,30]. A multivariate normal distribution was used to impute missing values of weight, height, smoking status, cholesterol, HDL, systolic blood pressure and HbA_1c_. In the UKPDS outcomes model, the required ethnicity values were White Caucasian, Afro-Caribbean and Asian–Indian. There were some unknown or unclassifiable values, e.g. mixed White + African, mixed White + Asian in ADDITION-UK. It was not suitable to replace these using multiple imputation, so we excluded these participants from the analysis (*n* = 25). After imputation, if the imputed HDL value was higher than the cholesterol value (which was logically impossible) we assumed that HDL = cholesterol – 0.1 (five cases at baseline; one case at five-year follow-up). Five imputations were taken for each participant and we combined results with Rubin's rules [31,32].

Using the UKPDS outcomes model we performed a patient-level modelling analysis on time horizons of 10, 20 and 30 years with a discount rate of 3.5%. We report the 30 years simulation as the main result. For each time horizon, 1,000 inner loops and 100 bootstraps were conducted. Means and confidence intervals at the patient level were used to conduct a further bootstrap analysis, adjusting for centre, age at diagnosis, gender and HbA_1c_ at baseline as per the short-term analysis. Incremental cost-effectiveness ratios (ICER) for the 10-, 20- and 30-year simulations were reported.

Decision uncertainty is illustrated with a scatter plot of incremental cost–QALY pairs and the cost-effectiveness acceptability curve (CEAC) [34]. One-way sensitivity analyses were performed on treatment costs (± 10%), utility decrements (± 10%) and the discount rate (0%, 5%) using the 30-year simulation data with the results shown as a tornado diagram [35]. We also explored two scenarios with an intervention cost of £750 (∼ 3/4 cost) and £500 (∼ 1/2 cost) to represent lower set-up costs (e.g. making use of previously designed materials).

Statistical analyses of within-trial data were performed using Statistics Analysis System (SAS, version 9.3). Analysis of long-term modelled scenarios was conducted using the UKPDS model and Microsoft Excel. This manuscript was prepared according to the Consolidated Health Economic Evaluation Reporting Standards (CHEERS) guidelines [36].

## Results

Among 1026 participants in ADDITION-UK trial, 2 people withdrew and 25 were excluded from the modelling analysis due to unknown or unclassifiable ethnicity. Thus, 1024 individuals were included in the short-term cost-effectiveness analysis and 999 individuals in the long-term modelling analysis.

Baseline characteristics of the study groups were well matched (Table 3). Mean age at diagnosis was slightly higher and mean cholesterol slightly lower in the intensive treatment group compared with the routine care group. The proportion of participants with a Caucasian ethnicity was higher in the intensive group.

**Table 3:** Baseline characteristics of the ADDITION-UK trial cohort

	Routine care group	Intensive treatment group
*N*	511	513
Mean age (sd), years	60.1 (7.5)	61.1 (7.2)
Female sex, %	40.7	36.6
Caucasian ethnicity, %	86.7	91.8
Current smoker, %	18.0	17.7
Mean BMI (sd), kg/m^2^	33.0 (5.9)	33.1 (5.6)
Mean total cholesterol (sd), mmol/l	5.5 (1.2)	5.3 (1.1)
Mean HDL (sd), mmol/l	1.2 (0.3)	1.2 (0.4)
Mean systolic blood pressure (sd), mmHg	143.1 (19.4)	142.0 (20.1)
Mean HbA_1c_ (sd), %	7.3 (1.7)	7.3 (1.7)

The total cost of delivering the intensive treatment intervention in ADDITION-UK over 5 years was £502 974, equating to a cost per person of £981 (£339 for materials and preparatory meetings, £370 for extra patient consultations and £262 for extra treatments). Cost by centre was £412 595 (£913 per person) in Cambridge and £90 379 (£1482 per person) in Leicester.

After a mean of 5 years (sd 1.1) of follow-up, there were large increases in the prescription of cardioprotective treatment and small, but significant, improvements in cardiovascular risk factors in favour of the intervention group (data not shown). The incidence of first cardiovascular event was 7.2% (13.5 per 1000 person-years) in the intensive treatment group and 8.5% (15.9 per 1000 person-years) in the routine care group [hazard ratio (HR) 0.83, 95% confidence interval (CI) 0.65 to 1.05] [12].

### Short-term cost-effectiveness analysis

There were no statistically significant differences in cumulative QALYs over any time horizon from 1 to 5 years (Table 4). The cumulative incremental cost to the NHS (intervention cost and other expenditure incurred as a result of cardiovascular complications) ranged from £285.30 over a 1-year horizon to £934.90 over 5 years (discounted at 3.5%). Because intensive treatment was both more costly and led to virtually zero incremental health gain compared with routine care over the first 5 years, intensive treatment of people with screen-detected Type 2 diabetes was not cost-effective in the short-term.

**Table 4:** Cumulative cost and QALYs in years following diabetes diagnosis, adjusted by centre, age, gender and HbA_1c_

	Routine care			Intensive treatment						
Time Horizon	N	Mean cost, £ (SE)	Mean QALYs (SE)	N	Mean cost, £ (SE)	Mean QALYs (SE)	Adjusted incremental cost, £ (95% CI)	Adjusted incremental QALYs (95% CI)	ICER	P (ICER < £30k)†
1	511	537 (1)	0.779 (0.000)	513	826 (2)	0.778 (0.000)	285 (199, 371)	0.0000 (–0.002, 0.002)	ICER was infinite or IT group was dominated* at all years	
2	511	1139 (2)	1.531 (0.000)	513	1426 (2)	1.530 (0.000)	279 (151, 406)	0.0000 (–0.004, 0.004)		
3	511	1706 (3)	2.256 (0.000)	513	2299 (3)	2.254 (0.000)	578 (389, 768)	0.0000 (–0.006, 0.006)		
4	501	2239 (3)	2.955 (0.000)	509	3015 (4)	2.954 (0.000)	754 (532, 977)	–0.0012 (–0.009, 0.007)		
5	451	2804 (5)	3.631 (0.000)	455	3773 (5)	3.627 (0.000)	935 (654, 1216)	–0.0040 (–0.016, 0.008)		
10	501	6157 (20)	6.450 (0.138)	498	7436 (12)	6.400 (0.140)	1190 (1126, 1245)	0.014 (–0.001, 0.029)	82252	1.0%
20	501	11175 (31)	9.324 (0.243)	498	12684 (21)	9.157 (0.243)	1496 (1368, 1625)	0.043 (0.001, 0.085)	34934	36.9%
30	501	13181 (22)	10.076 (0.293)	498	14769 (29)	9.818 (0.290)	1745 (1564, 1929)	0.047 (0.001, 0.093)	37503	31.4%

Results for time horizon of 1–6 years based on within-trial data. Results for time horizon of 10, 20 and 30 years based on results extrapolated using UKPDS model.

* Dominated = mean cost was higher and mean QALYs lower in the intensive treatment group.

† Probability that the ICER is below £30 000 per QALY gained.

### Long-term cost-effectiveness analysis

**FIGURE 1:**
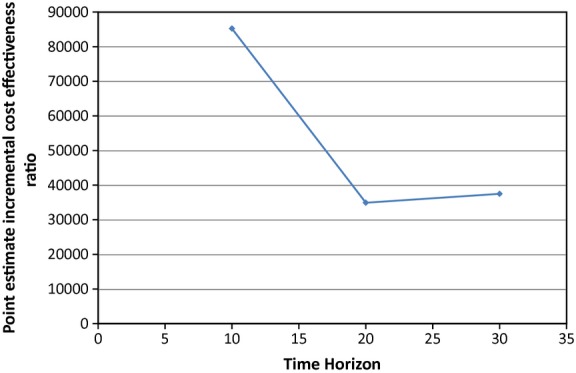
Broken line chart showing the simulated incremental cost-effectiveness ratios (ICERs) at 10, 20 and 30 years for the ADDITION-UK intervention.

Intensive treatment was associated with positive incremental QALYs (0.0465 by 30 years, statistically significant at 20 years and beyond). The incremental cost of intensive treatment versus routine care at 10, 20 and 30 years also increased over time to £1745 at 30 years, yielding point estimate ICERs of £82 250 at 10 years, falling to £35 000 at 20 years and increasing slightly to £37 500 at 30 years. The unadjusted results suggest a lower point estimate QALY gain in the intensive treatment arm, which is reversed once adjustment is made for baseline differences. The cumulative incidences of complications, death due to diabetes and to other-causes are reported in Table 5. At 30 years, there were trends towards reduced stroke, myocardial infarction, ischaemic heart disease, amputation, renal failure and death due to diabetes, but trends towards increased heart failure, blindness and death due to other causes. The result suggests that whilst cost-effectiveness improves over time, it is still above commonly accepted thresholds [26] even over a 30-year time horizon (Fig.1).

**Table 5:** Adjusted cumulative event incidence rates and adjusted risk factors from modelling simulation for 5, 10, 20, and 30 years

		Simulated years	Routine care	Intensive treatment	Adjusted difference* (Intensive treatment–Routine care)	Standard error
Complication	Stroke	5	0.0210	0.0226	−0.0009	0.0009
		10	0.0554	0.0589	−0.0023	0.0017
		20	0.1332	0.1396	−0.0041	0.0029
		30	0.1647	0.1700	−0.0041	0.0032
	Myocardial infarction	5	0.0667	0.0691	−0.0042	0.0023
		10	0.1495	0.1550	−0.0080	0.0042
		20	0.3125	0.3195	−0.0127	0.0070
		30	0.3748	0.3775	−0.0132	0.0077
	Ischaemic heart disease	5	0.0323	0.0329	−0.0012	0.0008
		10	0.0642	0.0651	−0.0019	0.0013
		20	0.1197	0.1202	−0.0018	0.0019
		30	0.1395	0.1380	−0.0018	0.0020
	Heart failure	5	0.0201	0.0210	−0.0005	0.0008
		10	0.0612	0.0635	−0.0011	0.0021
		20	0.1494	0.1545	0.0001	0.0040
		30	0.1851	0.1896	0.0017	0.0044
	Amputation	5	0.0027	0.0028	0.0001	0.0002
		10	0.0074	0.0072	−0.0002	0.0003
		20	0.0215	0.0210	−0.0002	0.0004
		30	0.0308	0.0291	−0.0005	0.0005
	Blindness	5	0.0183	0.0197	0.0002	0.0004
		10	0.0392	0.0418	0.0006	0.0005
		20	0.0734	0.0772	0.0013	0.0007
		30	0.0861	0.0889	0.0012	0.0007
	Renal failure	5	0.0015	0.0013	−0.0002	0.0001
		10	0.0047	0.0046	−0.0002	0.0002
		20	0.0156	0.0151	−0.0005	0.0004
		30	0.0227	0.0216	−0.0005	0.0006
	Diabetes death	5	0.0119	0.0134	0.0010	0.0018
		10	0.0416	0.0450	0.0003	0.0031
		20	0.1390	0.1457	−0.0019	0.0042
		30	0.1905	0.1950	−0.0017	0.0045
	Other death	5	0.0669	0.0710	−0.0029	0.0016
		10	0.1671	0.1776	−0.0049	0.0028
		20	0.4532	0.4779	−0.0019	0.0037
		30	0.7105	0.7240	0.0015	0.0051
Risk factor	HbA_1c_	5	7.33	7.30	−0.0060	0.0450
		10	8.09	8.07	−0.0116	0.0118
		20	8.74	8.74	−0.001	0.001
		30	9.04	9.04	−0.000	0.000
	Systolic blood pressure	5	138.59	137.38	−1.4835	0.7578
		10	141.98	141.38	−0.9157	0.5457
		20	143.76	143.32	−0.7630	0.5569
		30	144.43	143.99	−0.7575	0.5578

* Adjusted for age at diabetes diagnosis, sex, baseline HbA_1c_ and centre.

### Analysis of uncertainty

**FIGURE 2:**
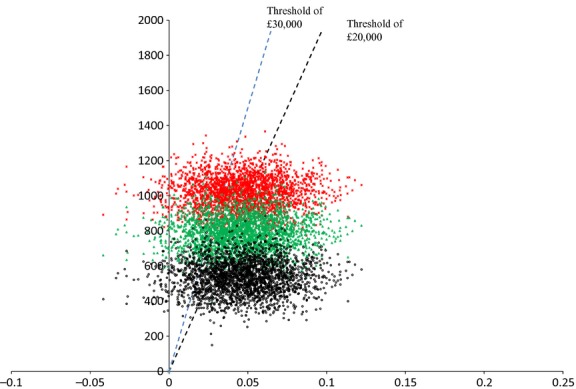
Cost-effectiveness plane showing pairs of incremental cost and QALYs from bootstrap samples using three different costs of delivering the intensive treatment intervention: £980.50 (red stars), £750 (green triangles) and £500 (black dots). The two dashed lines indicate the cost-effectiveness acceptability threshold of £20 000 (black line) and £30 000 (blue line). Points to the right of the lines are cost-effective.

Uncertainty and sensitivity analyses were performed on the predicted 30-year results. The cost-effectiveness plane based on bootstrap sampling (Fig.2) shows the scatter plot of cost and QALY pairs under the base case and two alternative scenarios with lower intervention cost. In the CE plane, we included three intervention costs: £981, £750 and £500. Under these scenarios, 30-year point estimate ICERs are £37 503, £32 550 and £27 178. The cost at which the ICER is £30 000 is £631. If the intervention could be delivered for this per patient or less, then the intervention may be considered cost-effective.

**FIGURE 3:**
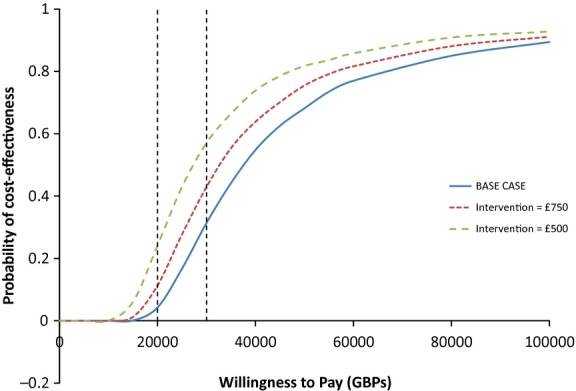
Cost-effectiveness acceptability curves which show the probability of intensive treatment being more cost-effective than routine care based on net benefit values from bootstrap samples using three different costs of delivering intensive treatment: £980.50 (blue), £750 (red) and £500 (green). The two dotted lines show the cost-effectiveness acceptability thresholds of £20 000 and £30 000 per QALY.

Under all three scenarios, the majority of points are in the NE quadrant, suggesting that the intensive treatment arm is nearly always both more expensive and more effective (generates more QALYs) than routine care, although the proportion of the probability mass in the NW quadrant suggests there is greater uncertainty around whether incremental QALYs are positive. The probability of cost-effectiveness according to the three different treatment costs is 51.1, 60.9 and 70.4% at a £20 000 threshold, and 65.1, 71.1 and 77.0% at £30 000 threshold respectively (Fig.3).

**FIGURE 4:**
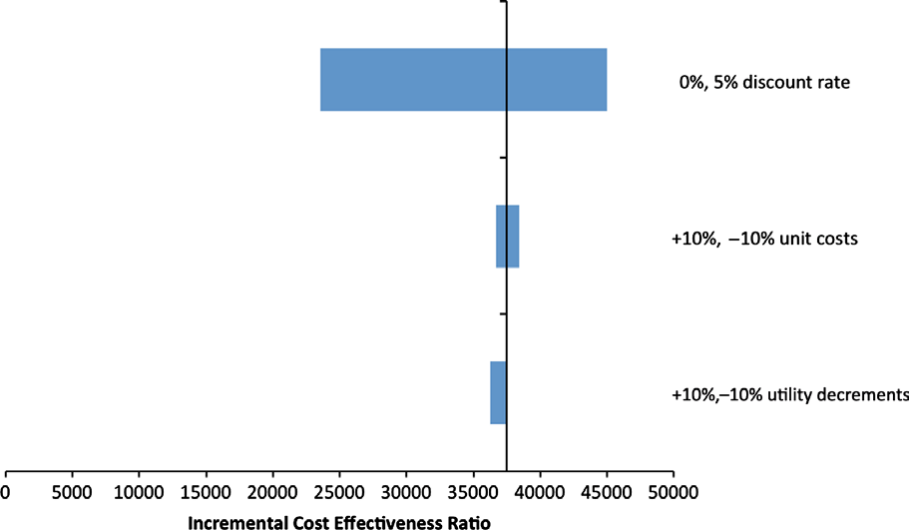
Tornado diagram showing the influence of changing different parameters that contribute to the ICER in long-term cost-effectiveness modelling analysis. Choice of discount rate has the greatest impact on the ICER (higher discount rate, unit costs and lower utility decrements all associated with higher point estimate ICER).

One-way sensitivity analyses varying unit treatment costs, utility decrements and discount rates showed that the discount rate had the biggest impact on the ICER, whereas the impact of utility decrements and treatment costs was minimal (Fig.4).

## Discussion

We report the short- and long-term cost-effectiveness of promotion of intensive multifactorial treatment compared to routine care for people with screen-detected Type 2 diabetes. Cumulative costs and QALYs over a time horizon of 1 to 5 years indicated that intensive treatment was not cost-effective in the short-term. This result may be linked to the finding that clinically important improvements in cardiovascular risk factors were observed in both study groups between baseline and follow-up [12]. Modest, but statistically significant differences between groups in the reduction of HbA_1c_, blood pressure and cholesterol favoured the intensive treatment group. The trial was undertaken during a time of improvements in the delivery of diabetes care in general practice, e.g. the introduction of the Quality and Outcomes Framework, which might have reduced the achievable differences in treatment and risk factors between groups. In the long-term modelling analysis, the 30 years simulated ICER was above the recommended UK NICE threshold (£20 000 to £30 000 per QALY) [26], suggesting that interventions to promote delivery of intensive multifactorial treatment in the ADDITION-UK study were not cost-effective compared with routine care in the long term.

The intervention only becomes cost-effective if it can be delivered at a cost of less than £631 per participant: we estimated the average cost per participant at £981 over 5 years. However, this figure may overestimate the cost to replicate the intervention because trial materials would not require complete redevelopment (although some adaptation to local needs would be likely) and alternative approaches to influencing practitioner behaviour, such as point-of-care reminders and decision aids, might be cheaper than practice visits by specialists. Furthermore, treatment costs were based on payments made to GP practices according to expected costs from the trial treatment algorithms. These costs may have been overestimated because GPs prescribed less medication than anticipated [12].

Our finding of a general improvement in cost-effectiveness over the long term is exemplary of the nature of preventive treatments. Because most of the cost of such interventions is borne ‘up front’, and chronic disease complications take a long time to develop, health and cost benefits are usually seen only in the long term. A key question for policy makers therefore is whether they are prepared to consider a longer time horizon in their decision-making.

A driver of our results compared with other studies might be the disease stage of study participants. The ADDITION study recruited participants detected by screening and therefore at an early stage in the disease trajectory. Early detection might help prevent complications in the future directly by attenuating disease progression, but intervening ‘too early’ might be less cost-effective. For example, intervening when a patient presents with diabetes-related symptoms may reduce the risk of an event that would otherwise have occurred in 5 years’ time. Intervening at an earlier stage, e.g. detection of impaired glucose tolerance, may prevent an event that would not otherwise occur for 20 years. Generally, individuals and society have a preference for benefits now rather than in the future, so would value preventing an event in five years’ time more highly than preventing that same event in 20 years.

### Strengths and limitations

This economic evaluation used data from a large randomized controlled trial and the cost-effectiveness analysis was conducted using a robust evaluation framework. We examined cost-effectiveness outcomes both for the short- and long-term and performed sensitivity analyses to test the robustness of our findings. We used data derived from a UK population with long-term CVD outcomes (the UKPDS study) to calculate unit costs, utility decrements and modelled CVD risk. We adjusted for centre (Cambridge/Leicester) rather than practice site, because the intervention differed slightly between the centres but within the same centre practices shared standardized practitioner guidelines. Intra-class correlation values for GP practice for the primary outcome were very small in the main ADDITION-Europe trial, supporting our decision to adjust for centre rather than practice.

There were some limitations. First, we only focused on macrovascular outcomes (CVD and associated acute events). Microvascular outcomes such as retinopathy and nephropathy are also important in assessing the impact of Type 2 diabetes on both quality of life and cost. Exclusion of these is likely to underestimate the benefit from any preventative intervention and thus underestimate the cost-effectiveness (that is, overestimate the ICER), particularly in the longer term as patients avoid cardiovascular disease and live with diabetes long enough to develop microvascular complications.

Second, although the treatment protocol was identical, the lifestyle intervention was different in Leicester and Cambridge. To represent the cost of implementing an ‘ADDITION-like’ intervention reflecting a degree of diversity across the country we simply averaged the two. We did, however, include centre in the adjusted analyses estimating incremental cost and QALYs. Results were similar when we adjusted for cluster (GP practice) and when running analyses separately by centre.

Third, because the results of this study were largely driven by data from Cambridge (*n* = 867 participants versus *n* = 159 in Leicester), where the vast majority of participants were Caucasian, the generalizability to other more ethnically diverse populations must be considered with caution. However, the capacity to benefit from intensive multifactorial treatment is probably higher given the increased risk of diabetes and diabetes-related complications in ethnic minority groups [39].

Fourth, we used an additive method to calculate treatment costs and utility decrements for individuals with multiple events. This is a commonly used method, which was applied in the UKPDS outcomes model [22,40], but it is unclear if the cost and utility decrement of subsequent complications should be additive or multiplicative.

Fifth, most of the equations in the UKPDS predict future risk factors and events based on baseline values, time since diagnosis and the value of biometrics such as HbA_1c_ in the previous period (year). It is an individual-level model in which patient data are calculated individually (rather than as mean values of a cohort). Thus, the model assumes that any trends present at 5 years will continue into the future, effectively assuming a continuation of the effect of the small differences in treatment generated by the intervention at 5 years. Ten-year follow-up of the UKPDS cohort found that although between-group differences in HbA_1c_ and blood-pressure were both lost within 12 months of the end of the active phase of the study, those who had previously achieved tighter control over HbA_1c_ still had a lower event rate at 10 years than those who had not, whereas any benefit from a lower blood pressure was not maintained [41,42]. Current guidelines and practice have changed since the initiation of the *ADDITION* trial, with patients recommended to receive at least as intensive treatment as the ‘intensive’ arm. Trial patients in the routine care arm received a higher level of treatment than observed in ‘standard practice’, which may have diluted the observed incremental health gain. However, after 5 years of treatment, there was room for improvement in the prescription of cardioprotective treatment in both groups. Current evidence suggests that delays in treatment intensification in people with Type 2 diabetes (clinical inertia) are still very common.

On the cost side, the official duration of the intensive intervention was 3 years, for which practices were reimbursed for additional activity and prescriptions. We projected additional prescribed drug costs in the intervention arm over the full 30 years, under the assumption that patients in the control arm would not increase their medication, and patients in the intervention arm would not decrease their medication over time. We may, therefore, have overestimated the cost of the intervention arm. Whether this is true or not will be assessed once the 10-year follow-up data of the ADDITION cohort are available for analysis.

Finally, although we used the most appropriate CVD risk model available, the UKPDS outcomes model was derived using data from an historical cohort of people with clinically diagnosed diabetes. The ADDITION cohort included people with screen-detected diabetes. Other studies [43,44] show that the UKPDS outcomes model tends to overestimate absolute CVD risk, a finding replicated in our own investigation of the suitability of the UKPDS to extrapolate ADDITION data [28]. This is unsurprising because the UKPDS cohort collected data between 1977 and 1997, and the treatment of diabetes and its complications has improved substantially, leading to changes in treatment costs and outcomes. However, for the prediction of differences between intervention groups, the results of our validation analysis [28] were mixed: the UKPDS model overestimated the group difference for stroke but underestimated the difference for myocardial infarction, albeit within ‘tolerable’ limits. In addition, the utility decrements for myocardial infarction and stroke derived from the ADDITION-UK study were smaller than those in UKPDS, a finding consistent with contemporary patients receiving better care and hence reporting higher quality of life. This underlines the importance of our sensitivity analyses, showing the robustness of the results to changes in the input parameters. The discount rate has the biggest impact compared with the intervention unit cost and utility decrement.

In conclusion, promotion of intensive multifactorial treatment compared to routine care for people with screen-detected Type 2 diabetes does not appear to be cost-effective in the ADDITION-UK study. However, the intervention has the potential to be cost-effective if it can be delivered for approximately £630 per patient rather than £981. Such savings may be plausible through adaptation of pre-developed materials and economies of scale in delivery.

## Supporting Information

**Table S1.** Baseline and five-year follow-up values for clinical variables, separately by trial group in the ADDITION-UK trial cohort; all values are mean (sd). **Table S2.** Sensitivity analysis for the short term: ± 10% unit treatment costs, ± 10% utility decrements, and 0% and 5% discount rate. **Table S3.** Sensitivity analysis for long term: ± 10% unit treatment costs, ± 10% utility decrements, and 0% and 5% discount rate.

## References

[1] Arredondo A (2013). Diabetes: a global challenge with high economic burden for public health systems and society. Am J Public Health.

[2] Liu S, Zhao Y, Hempe JM, Fonseca V, Shi L (2012). Economic burden of hypoglycemia in patients with Type 2 diabetes. Expert Rev Pharmacoecon Outcomes Res.

[3] McInnes AD (2012). Diabetic foot disease in the United Kingdom: about time to put feet first. Journal Foot Ankle Res.

[4] Idris I (2009). New NICE guideline for managing hyperglycaemia in patients with type 2 diabetes recognised the use of newer therapies. Diabet Obes Metab.

[5] Mathiesen ER, Damm P (2008). Commentary from Copenhagen on the NICE guideline on management of diabetes and its complications from preconception to the postnatal period. Diabet Med.

[6] Walker JD (2008). NICE Guidance on Diabetes in Pregnancy: Management of Diabetes and its Complications from Preconception to the Postnatal Period.

[7] Gaede P, Valentine WJ, Palmer AJ, Tucker DM, Lammert M, Parving HH (2008). Cost-effectiveness of intensified versus conventional multifactorial intervention in type 2 diabetes: results and projections from the Steno–2 study. Diabetes Care.

[8] Gaede P, Vedel P, Larsen N, Jensen GV, Parving HH, Pedersen O (2003). Multifactorial intervention and cardiovascular disease in patients with type 2 diabetes. N Engl J Med.

[9] Khunti K, Morris DH, Weston CL, Gray LJ, Webb DR, Davies MJ (2013). Joint prevalence of diabetes, impaired glucose regulation, cardiovascular disease risk and chronic kidney disease in South Asians and White Europeans. PloS one.

[10] UK National Screening Committee (2013). The UK NSC Policy on Diabetes Screening in Adults.

[11] Lauritzen T, Griffin S, Borch-Johnsen K, Wareham NJ, Wolffenbuttel BH, Rutten G (2000). The ADDITION study: proposed trial of the cost-effectiveness of an intensive multifactorial intervention on morbidity and mortality among people with Type 2 diabetes detected by screening. Int J Obes Relat Metab Disord.

[12] Griffin SJ, Borch-Johnsen K, Davies MJ, Khunti K, Rutten GE, Sandbaek A (2011). Effect of early intensive multifactorial therapy on 5-year cardiovascular outcomes in individuals with type 2 diabetes detected by screening (ADDITION-Europe): a cluster-randomised trial. Lancet.

[13] McIntosh AHA, Home PD, Brown F, Bruce A, Damerell A, Davis R (2001). Clinical guidelines and evidence review for Type 2 diabetes: management of blood glucose.

[14] McIntosh AHA, Home PD, Brown F, Bruce A, Damerell A, Davis R (2002). Clinical guidelines and evidence review for Type 2 diabetes: management of blood pressure.

[15] McIntosh AHA, Home PD, Brown F, Bruce A, Damerell A, Davis R (2002). Clinical guidelines and evidence review for Type 2 diabetes: lipids management.

[16] Davies MJ, Heller S, Skinner TC, Campbell MJ, Carey ME, Cradock S (2008). Effectiveness of the diabetes education and self management for ongoing and newly diagnosed (DESMOND) programme for people with newly diagnosed type 2 diabetes: cluster randomised controlled trial. BMJ.

[17] Heart Outcomes Prevention Evaluation Study Investigators (2000). Effects of ramipril on cardiovascular and microvascular outcomes in people with diabetes mellitus: results of the HOPE study and MICRO-HOPE substudy. Lancet.

[18] UK Prospective Diabetes Study UKPDS) Group (1998). Intensive blood-glucose control with sulphonylureas or insulin compared with conventional treatment and risk of complications in patients with type 2 diabetes (UKPDS 33. Lancet.

[19] Collins R, Armitage J, Parish S, Sleigh P, Peto R (2003). Heart Protection Study Collaborative G. MRC/BHF Heart Protection Study of cholesterol-lowering with simvastatin in 5963 people with diabetes: a randomised placebo-controlled trial. Lancet.

[20] Curtis L (2004). PSSRU Inflation Indices, Secondary PSSRU Inflation Indices.

[21] Curtis L (2010). Unit Costs of Health and Social Care 2010.

[22] Clarke P, Gray A, Legood R, Briggs A, Holman R (2003). The impact of diabetes-related complications on healthcare costs: results from the United Kingdom Prospective Diabetes Study (UKPDS Study No. 65). Diabet Med.

[23] Clarke PM, Gray AM, Briggs A, Farmer AJ, Fenn P, Stevens RJ (2004). A model to estimate the lifetime health outcomes of patients with type 2 diabetes: the United Kingdom Prospective Diabetes Study (UKPDS) Outcomes Model (UKPDS no. 68). Diabetologia.

[24] Clarke P, Gray A, Holman R (2002). Estimating utility values for health states of type 2 diabetic patients using the EQ-5D (UKPDS 62). Med Decis Making.

[25] Szende A, Oppe M, Devlin N (2007). EQ–5D Value Sets: Inventory, Comparative Review and User Guide.

[26] National Institute for Health and Care Excellence (2013). Guide to the Methods of Technology Appraisal 2013.

[27] Eriksson KF, Lindgarde F (1991). Prevention of type 2 (non-insulin-dependent) diabetes mellitus by diet and physical exercise. The 6-year Malmo feasibility study. Diabetologia.

[28] Tao L, Wilson EC, Griffin SJ, Simmons RK, ADDITION-Europe Study Team (2013). Performance of the UKPDS outcomes model for prediction of myocardial infarction and stroke in the ADDITION-Europe trial cohort. Value Health.

[29] Rubin DB, Schenker N (1991). Multiple imputation in health-care databases: an overview and some applications. Stat Med.

[30] Yuan Y (2011). Multiple Imputation Using SAS Software. J Stat Software.

[31] Rubin DB, Schenker N (1991). Multiple imputation in health-care databases: an overview and some applications. Stat Med.

[32] Rubin DB (2004). Mutliple Imputation for Nonresponse in Surveys.

[33] Efron B (2011). The bootstrap and Markov-chain Monte Carlo. J Biopharm Stat.

[34] Fenwick E, Byford S (2005). A guide to cost-effectiveness acceptability curves. Br J Psychiat.

[35] Eschenbach TG (2006). Technical Note: Constructing tornado diagrams with spreadsheets. Eng Econ.

[36] Husereau D, Drummond M, Petrou S, Carswell C, Moher D, Greenberg D (2013). Consolidated Health Economic Evaluation Reporting Standards (CHEERS) statement. BMJ.

[37] Gillett M, Dallosso HM, Dixon S, Brennan A, Carey ME, Campbell MJ (2010). Delivering the diabetes education and self management for ongoing and newly diagnosed (DESMOND) programme for people with newly diagnosed type 2 diabetes: cost effectiveness analysis. BMJ.

[38] Drummond M, Sculpher M, Torrance G, O'Brien B, Stoddart G (2005). Methods for the Economic Evaluation of Health Care Programmes.

[39] Gholap N, Davies M, Patel K, Sattar N, Khunti K (2011). Type 2 diabetes and cardiovascular disease in South Asians. Primary Care Diabet.

[40] Brandle M, Zhou H, Smith BR, Marriott D, Burke R, Tabaei BP (2003). The direct medical cost of type 2 diabetes. Diabetes Care.

[41] Holman RR, Paul SK, Bethel MA, Matthews DR, Neil HA (2008). 10-year follow-up of intensive glucose control in type 2 diabetes. N Engl J Med.

[42] Holman RR, Paul SK, Bethel MA, Neil HA, Matthews DR (2008). Long-term follow-up after tight control of blood pressure in type 2 diabetes. N Engl J Med.

[43] Coleman RL, Stevens RJ, Retnakaran R, Framingham Holman RR (2007). SCORE, and DECODE risk equations do not provide reliable cardiovascular risk estimates in type 2 diabetes. Diabetes Care.

[44] Kengne AP, Patel A, Colagiuri S, Heller S, Hamet P, Marre M (2010). The Framingham and UK Prospective Diabetes Study (UKPDS) risk equations do not reliably estimate the probability of cardiovascular events in a large ethnically diverse sample of patients with diabetes: the Action in Diabetes and Vascular Disease: Preterax and Diamicron-MR Controlled Evaluation (ADVANCE) Study. Diabetologia.

[45] Schwarz B, Gouveia M, Chen J, Nocea G, Jameson K, Cook J (2008). Cost-effectiveness of sitagliptin-based treatment regimens in European patients with type 2 diabetes and haemoglobin A1c above target on metformin monotherapy. Diabet Obes Metab.

[46] Valentine WJ, Bottomley JM, Palmer AJ, Brandle M, Foos V, Williams R (2007). PROactive 06: cost-effectiveness of pioglitazone in Type 2 diabetes in the UK. Diabet Med.

