# Supplementary material for: Cost-effectiveness of intensive multifactorial treatment compared with routine care for individuals with screen-detected Type 2 diabetes: analysis of the ADDITION-UK cluster-randomized controlled trial
Source: Diabet Med. 2015 Apr 15;32(7):907–19. doi: 10.1111/dme.12711 (PMC4510785; doi:10.1111/dme.12711)
Supplement: Supplementary file 1 [file dme0032-0907-sd1.doc]

**Table S1.** Baseline and five-year follow-up values for clinical variables, separately by trial group in the *ADDITION-UK* trial cohort; all values are mean (SD)

|  | Routine care group | | | Intensive treatment group | | |
| --- | --- | --- | --- | --- | --- | --- |
|  | Baseline | Follow up | Adjusted difference*  (follow up-baseline)  (SE) | Baseline | Follow up | Adjusted difference*  (follow up-baseline)  (SE) |
| HbA_1c_ (%) | 7.3 (1.7) | 7.1 (1.0) | –0.25 (0.09) | 7.3 (1.7) | 6.9 (1.0) | –0.37 (0.09) |
| Total cholesterol  (mmol/l) | 5.5 (1.2) | 4.3 (0.9) | –1.20 (0.07) | 5.3 (1.1) | 4.0 (0.9) | –1.30 (0.06) |
| Systolic blood pressure  (mmHg) | 143.1 (19.4) | 136.2 (15.6) | –7.08 (1.13) | 142.0 (20.1) | 134.4 (16.7) | –7.32 (1.18) |

* Adjusted for age at diabetes diagnosis, sex and centre

**Table S2.** Sensitivity analysis for short term: ±10% unit treatment costs, ±10% utility decrements, and 0% 5% discount

| Follow up years |  | –10% cost | +10% cost | –10% utility | +10% utility | 0% discount | | 5% discount | |
| --- | --- | --- | --- | --- | --- | --- | --- | --- | --- |
|  | N | Incremental treatment cost | | Incremental QALYs | | Incremental cost | Incremental QALYs | Incremental cost | Incremental QALYs |
| 1 | 511 | 44.2 | 53.9 | –0.001 | –0.010 | 49.1 | –0.001 | 49.1 | –0.001 |
| 2 | 511 | –43.4 | –53.0 | –0.001 | –0.001 | –51.6 | –0.001 | –46.8 | –0.001 |
| 3 | 511 | 8.7 | 10.6 | –0.001 | –0.001 | 10.3 | –0.001 | 9.4 | –0.001 |
| 4 | 501 | 5.7 | 6.9 | –0.001 | –0.002 | 5.4 | –0.002 | 6.6 | –0.002 |
| 5 | 451 | 9.6 | 11.7 | –0.004 | –0.005 | 5.5 | –0.005 | 12.6 | –0.005 |

**Table S3.** Sensitivity analysis for long term: ±10% unit treatment costs, ±10% utility decrements, and 0% 5% discount

|  |  | Incremental treatment cost | Incremental QALYs | ICER |
| --- | --- | --- | --- | --- |
| Unit treatment costs | –10% | 1730.26 | 0.0572 | 36681.22 |
|  | +10% | 1730.53 | 0.0451 | 38402.13 |
|  |  |  |  |  |
| Utility decrements | –10% | 1735.59 | 0.0468 | 37047.05 |
|  | +10% | 1745.90 | 0.0481 | 36270.71 |
|  |  |  |  |  |
| Discount rates | 0% | 1769.04 | 0.0751 | 23557.08 |
|  | 5% | 1738.94 | 0.0387 | 44952.77 |
